# Transdiagnostic Psychiatric Symptoms and Event-Related Potentials following Rewarding and Aversive Outcomes

**DOI:** 10.1371/journal.pone.0157084

**Published:** 2016-06-14

**Authors:** Jeffrey S. Bedwell, Geoffrey F. Potts, Diane C. Gooding, Benjamin J. Trachik, Chi C. Chan, Christopher C. Spencer

**Affiliations:** 1 Department of Psychology, University of Central Florida, Orlando, Florida, United States of America; 2 Department of Psychology, University of South Florida, Tampa, Florida, United States of America; 3 Department of Psychology, University of Wisconsin-Madison, Madison, Wisconsin, United States of America; University of Medicine & Dentistry of NJ - New Jersey Medical School, UNITED STATES

## Abstract

There is a need for a better understanding of transdiagnostic psychiatric symptoms that relate to neurophysiological abnormalities following rewarding and aversive feedback in order to inform development of novel targeted treatments. To address this need, we examined a transdiagnostic sample of 44 adults (mean age: 35.52; 57% female), which consisted of individuals with broadly-defined schizophrenia-spectrum disorders (n = 16), bipolar disorders (n = 10), other mood and anxiety disorders (n = 5), and no history of a psychiatric disorder (n = 13). Participants completed a Pavlovian monetary reward prediction task during 32-channel electroencephalogram recording. We assessed the event-related potentials (ERPs) of feedback-related negativity (FRN), feedback-related positivity (FRP), and the late positive potential (LPP), following better and worse than expected outcomes. Examination of symptom relationships using stepwise regressions across the entire sample revealed that an increase in the clinician-rated Negative Symptoms factor score from the Positive and Negative Syndrome Scale, was related to a decreased LPP amplitude during better than expected (i.e., rewarding) outcomes. We also found that increased self-reported scores on the Schizotypal Personality Questionnaire (Brief-Revised) Disorganized factor related to an increased FRN amplitude during worse than expected (i.e., aversive) outcomes. Across the entire sample, the FRP component amplitudes did not show significant relationships to any of the symptoms examined. Analyses of the three diagnostic groups of schizophrenia-spectrum disorders, bipolar disorders, and nonpsychiatric controls did not reveal any statistically significant differences across the ERP amplitudes and conditions. These findings suggest relationships between specific neurophysiological abnormalities following rewarding and aversive outcomes and particular transdiagnostic psychiatric symptoms.

## Introduction

Abnormalities in reward processing have been reported across a number of psychiatric disorders and may relate to particular transdiagnostic symptoms theoretically related to reward such as anhedonia and avolition [1–4]. Researchers have recently highlighted the need for a better understanding of neurophysiological abnormalities during different stages of reward processing to inform the development of new treatments that can be targeted to related abnormalities [1, 5]. Recent reviews have indicated that although consummatory reward processes appear intact in depression and schizophrenia [1, 5], dopaminergic signaling in the striatum involved with reinforcement learning is reduced in major depressive disorder and schizophrenia, but elevated in bipolar disorder [1]. It is possible that biologically-informed treatments may have differential efficacy when used across different psychiatric disorders. For example, based on this literature, a dopaminergic agonist such as bupropion may improve the blunted reinforcement learning for some individuals with depression, but may aggravate the same abnormality in some individuals with bipolar disorder. As individuals with a given disorder vary on the severity of symptoms involving reward (e.g., anhedonia, avolition), a better understanding of how these reward-system abnormalities relate to particular symptom severity, rather than disorder category, will likely lead to greater predictive power in patient-level treatment-matching efforts. Similarly, psychosocial treatments such as behavioral activation therapy [6, 7] may be more successful with individuals with particular symptoms [8], regardless of the disorder, that relate to abnormalities in an aspect of reward processing [7]. Thus, increased knowledge about transdiagnostic symptoms related to reward processing abnormalities can directly inform ongoing efforts to tailor treatment according to symptom severity patterns.

The processing of rewarding and aversive outcomes is not a single function but rather a sequential series of cognitive operations which includes anticipatory, consummatory, and learning components [9]. Examination of event-related potentials (ERPs) is a useful method to investigate distinct transient cognitive processes that may occur temporally adjacent to one another, and when the exact neural substrates are less important to the study’s goals. One such ERP component, the feedback-related negativity (FRN; also referred to as medial frontal negativity, feedback negativity, or feedback error-related negativity) is a negative voltage deflection over mediofrontal electrode sites that peaks around 200 to 300 ms, and is strongest after a worse than expected (i.e., aversive) outcome [10, 11]. A related ERP, feedback-related positivity (FRP; also referred to as P2a or reward positivity), is a positive voltage deflection following a better than expected (i.e., rewarding) outcome over the same electrode sites and time course [10, 12, 13]. Some researchers do not observe a separate FRP component in the positive voltage range, and instead refer to a reduction in the FRN amplitude to reflect what we and others refer to as FRP. These components are thought to reflect phasic decreases (FRN) or increases (FRP) in the same midbrain dopamine outcome-to-expectation evaluation system [14, 15]. The different terminology used to describe these components is further complicated by use of difference waves (e.g., non-reward minus reward) in some, but not all, research groups.

In contrast, the late positive potential (LPP) component is a more sustained positive voltage deflection following emotional stimuli (e.g., monetary gain/loss feedback; emotionally-evocative pictures) [16, 17]. The LPP begins around 350 ms after stimulus onset, is maximal over centroparietal electrodes, and often continues for the duration of the stimuli as well as a variable period after stimulus offset [16]. The average LPP voltage following either positively or negatively valenced stimuli is larger than the response to neutral stimuli [18–20]. The literature is mixed with regard to whether positive or negative stimuli elicit a larger LPP (see [19]). It appears that when positive stimuli are similarly motivationally/biologically salient (e.g., erotic and affiliative pictures) as the negative stimuli (e.g., mutilated bodies), there does not appear to be a difference in the respective LPP magnitudes [19, 21]. The FRN/FRP may reflect the immediate updating of expectations following an outcome that did not meet reward expectation, while the LPP may reflect more sustained emotional processing of that unexpected outcome. By examining all three components, the current study is able to deconstruct particular aspects of reward processing that relate to specific transdiagnostic symptoms. This could then inform both psychosocial and pharmacological treatments that could focus on the particular stage and network of reward processing that is related to a distinct psychiatric symptom (e.g., avolition), independent of the formal diagnosis. The current study also separately examines better and worse than expected outcomes, as abnormal ERP responses may be specific to rewarding vs. aversive outcomes, which is also highly relevant to treatment approaches.

In the psychiatric literature, existing studies have focused on these ERP components in a single psychiatric disorder sample as compared to nonpsychiatric controls, and have rarely examined symptom relationships. As our sample was primarily composed of individuals with schizophrenia-spectrum and bipolar disorders, we examined findings from studies assessing similar disorders or related symptoms. Two studies examined FRN in schizophrenia samples using simple gambling paradigms and both found no difference in a FRN difference waveform amplitude (non-reward minus reward) between individuals with schizophrenia and nonpsychiatric controls [22, 23]. It appears that there is only one published study examining FRN in euthymic bipolar disorder which found a reduced FRN amplitude to positive outcomes, suggesting a positive evaluation bias as the reduced FRN is analogous to a larger FRP to reward [24]. The same research group also found an attenuated FRN to both better and worse than expected outcomes [25], and to immediate vs. delayed rewards [26], in nonpsychiatric adults at risk for hypomania, suggesting that the positive evaluation bias extends to this related subclinical population.

In terms of the LPP, findings in schizophrenia samples are mixed. Two studies reported reduced LPP amplitude to positively-valenced stimuli in individuals with schizophrenia [27, 28], but two others found no difference [29, 30]. Similarly, two studies reported reduced LPP amplitude to negatively-valenced stimuli in individuals with schizophrenia [31, 32], but four others reported no difference [28–30, 33]. One study that examined symptom relationships to the LPP in schizophrenia found that the reduction in LPP amplitude to negative pictures related to an increase in negative symptom severity [31]; however, this study did not examine LPP response to positive pictures. At least two other studies did not find a relationship between LPP amplitude and broad symptom categories in schizophrenia [28, 30]. There does not appear to be any published research examining the LPP in relation to emotional stimuli in individuals with bipolar disorder or related subclinical conditions.

The current study builds on this existing literature with a broad transdiagnostic examination of both self-reported and clinician-rated psychiatric symptoms in relation to the three ERP components elicited during a Pavlovian monetary reward conditioning task. In order to examine whether any ERP differences were specific to a diagnostic group, we complemented the transdiagnostic approach with pairwise diagnostic class comparisons between schizophrenia-spectrum disorders, bipolar disorders, and nonpsychiatric controls. Finally, the current study examines FRN/FRP components to clarify the relative response to worse or better than expected outcomes respectively, in addition to the more sustained emotional processing as indexed by the LPP component. Considering the limited and mixed findings in individual psychiatric disorders, and the lack of existing research using a transdiagnostic approach, the study is primarily exploratory in nature. However, based on the limited literature and theoretical considerations, we hypothesized that the severity of negative symptoms would relate to a reduced sustained processing of emotional stimuli, as indexed by the LPP, but not relate to the immediate conditioning and attentional aspects indexed by the FRP and FRN. In addition, based on initial findings in bipolar disorder and the trait of hypomania, we hypothesized that the pairwise diagnostic comparisons would reveal an enhanced FRP (analogous to attenuated FRN found in previous studies) for the bipolar disorder group when compared to both other groups.

## Materials and Methods

### Participants

Participants were recruited from the local community in a manner that yielded a wide range of psychiatric disorders, with a directed effort in recruiting individuals with schizophrenia-spectrum and bipolar disorders, as these conditions often include reward processing deficits based on a variety of behavioral, physiological, and neuroimaging methodologies. These disorders were also the focus of a broader study that included this task. Participants were recruited using a combination of newspaper advertisements, Craigslist postings, flyers placed at psychiatric facilities, and word-of-mouth. These advertisements requested participation from individuals diagnosed with “schizophrenia, schizoaffective disorder, or bipolar disorder.” Other advertisements did not mention a psychiatric disorder and were intended to primarily recruit nonpsychiatric adults. Our independent diagnostic evaluation yielded a wider range of disorders from both of these types of advertisements. All participants completed informed consent and received a cash stipend of $16 per hour. Participants, including those with no psychiatric diagnosis, were excluded if they reported a history of significant neurological symptoms, recent substance abuse or dependence, medical conditions that may affect brain functioning, and English not being the native language. In addition, we administered the Reading subtest from the Wide Range Achievement Test 3^rd^ edition (WRAT-3; [34]) and excluded participants with estimated IQ < 70, as well as individuals with an estimated corrected visual acuity worse than 20/40 using a Snellen wall chart.

Following these exclusions, 52 participants completed the study. However, eight of these individuals had to be excluded for poor accuracy on the task (described below), resulting in a final sample of 44 individuals who were included in data analyses (57% female; mean age = 35.52, SD = 8.94; range = 19 to 55). Diagnoses were based on administration of the Structured Clinical Interview for DSM-IV Axis-I Disorders (SCID-I) and the Avoidant, Paranoid, and Schizotypal (schizophrenia-spectrum) sections of the Structured Clinical Interview for DSM-IV Axis-II Disorders (SCID-II) by two clinical psychology doctoral students (authors BJT and CCC). Consensus diagnoses were formed after case presentations with a licensed clinical psychologist (author JSB). For one set of analyses, we examined three broad diagnostic classes of interest: 1) schizophrenia-spectrum disorders (n = 16; 5 schizophrenia, 5 schizoaffective disorder, 2 delusional disorder, 2 paranoid personality disorder, 1 schizotypal personality disorder, 1 avoidant personality disorder); 2) bipolar disorder (n = 10; 9 bipolar I disorder [6 of which had history of psychosis] and 1 bipolar II disorder); and 3) no psychiatric disorder (n = 13). There were no significant differences in distributions of sex, age, or overall task accuracy across these three groups (all *p*s > .15). For analyses that examined symptom relationships across entire sample, we included an additional five participants with other mood and anxiety disorders (2 social phobia, 1 dysthymic disorder, 1 generalized anxiety disorder, 1 posttraumatic stress disorder). See Table 1 for demographic, cognitive, and clinical characteristics across the entire sample.

**Table 1:** Descriptive Statistics of Demographic, Cognitive, and Clinical Variables.

Sample Size	44
Age	35.52 (8.94); 19–55
Sex (% Female)	57%
Race (% Non-Hispanic White)	66%
Estimated IQ*	102.35 (10.67); 72–117
Years of Education	14.48 (2.54); 7–20
PANSS Positive Symptoms	12.32 (5.45); 7–27
PANSS Negative Symptoms	12.64 (5.62); 7–28
PANSS Depression Item (G6)	2.93 (1.34); 1–6
PANSS Anxiety Item (G2)	2.11 (1.15); 1–5
SPQ-BR Cognitive-Perceptual Factor	15.23 (10.11); 2–42
SPQ-BR Interpersonal Factor	11.65 (7.26); 0–27
SPQ-BR Disorganized Factor	16.70 (7.39); 0–28
ACIPS Total Score	77.98 (16.02); 26–102
TEPS Total Score	80.27 (12.51); 39–100

Unless otherwise noted, descriptive data are in the format of [mean (standard deviation); range].

* IQ was estimated using the standard score from the Reading subtest of the Wide Range Achievement Test– 3^rd^ edition (missing data for one participant).

PANSS = Positive and Negative Syndrome Scale; SPQ-BR = Schizotypal Personality Questionnaire-Brief Revised (missing data from one participant); ACIPS = Anticipatory and Consummatory Interpersonal Pleasure Scale; TEPS = Temporal Experience of Pleasure Scale.

### Procedures

The study was approved by the university’s Institutional Review Board. Following administration of the diagnostic interviews and visual/cognitive screens, participants completed the Structured Clinical Interview for Positive and Negative Syndrome Scale (PANSS; [35]). In addition to the PANSS Positive and Negative Symptom factors, the current study also examined the Depression (Item G6) and Anxiety (Item G2) items from the General factor, as these represent broad transdiagnostic symptoms of interest. Participants also completed three self-report scales–the Schizotypal Personality Questionnaire–Brief Revised (SPQ-BR; [36, 37], the Temporal Experience of Pleasure Scale (TEPS; [38]), and the Anticipatory and Consummatory Interpersonal Pleasure Scale (ACIPS; [39, 40]). The SPQ-BR generates Cognitive-Perceptual, Interpersonal, and Disorganized factor scores [37]. The TEPS total score was chosen as a measure of general anhedonia, and the ACIPS total score as a measure of social anhedonia. In both scales, lower scores represent increased anhedonia. One participant was missing data from the SPQ-BR.

#### Reward Conditioning Task and EEG Acquisition

EEG data were collected and recorded using a Neuroscan Synamps2 system (32 channels). Data were acquired using DC recording, digitized at 1000 Hz, with a 100 Hz low pass filter and no high pass filter. Prior to EEG recording, impedance of less than 5 kOhms was obtained for each scalp electrode. The linked bilateral mastoid electrodes were used as the recording reference, and vertical and horizontal ocular electrodes were used to estimate blinks and large eye movements.

Participants completed a Pavlovian monetary reward conditioning task during EEG recording, which was closely modeled after the task used by Potts and colleagues [10], and later adopted by others in schizophrenia research [22]. Each session began with task instructions followed by practice trials. Participants were informed that a randomly selected winning task block would result in actual monetary awards received in addition to the advertised payment for research participation. Participants were also informed that they would begin each block with a negative balance of 7 dollars (which ensured that they would earn final winnings in the desired range), that each trial would “cost” 25 cents, and that each “win,” resulting from the second stimulus being a gold bar, would result in a monetary gain of 1 US dollar. Participants were told that sometimes the two stimuli would be identical and other times they would not match, but were not told about the related conditioning aspect or relative frequencies of these conditions. The participants completed four consecutive trial blocks, each containing 60 trials, for a total of 240 trials. The amount that the participants could “win” was preset for each given block, which ranged from $5 to $11.

**Fig 1:**
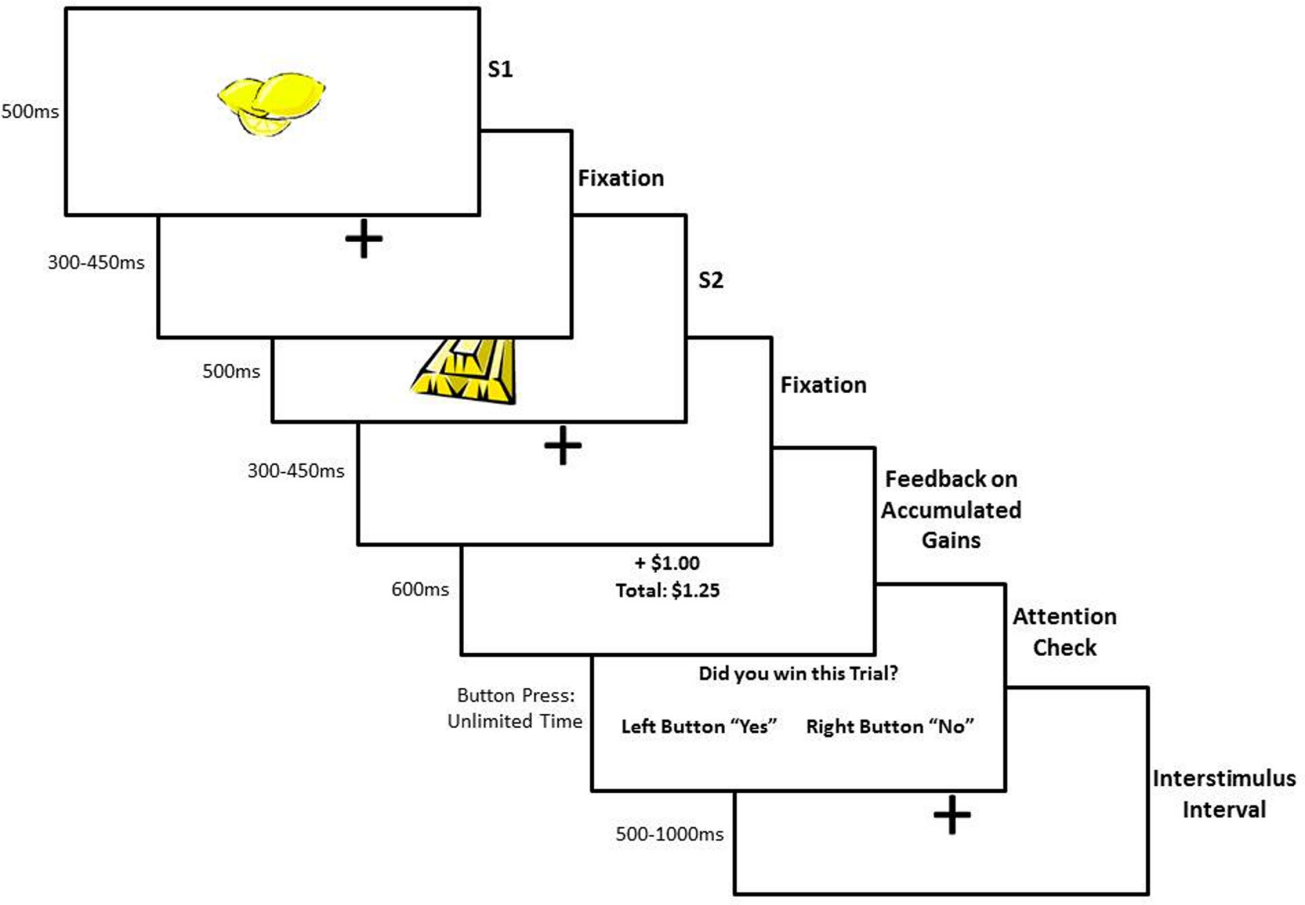
Depiction of the reward task design.

Each trial consisted of the initial S1 stimulus (picture of a lemon or gold bar) presented for 500 ms, followed by a randomly jittered fixation (picture of a cross in center) interstimulus interval (ranging from 300 ms—450 ms), before the onset of S2 (picture of a lemon or gold bar), which appeared for 500 ms. Following S2, another fixation of 300–450 ms appeared before a display of the running total of accumulated gains that was presented for 600 ms before an inter-trial interval (500–1000 ms) and the start of the next trial. See Fig 1 for visual depiction of task and stimuli. Participants were required to indicate whether they had “won” the previous trial using buttons on a handheld gaming device. The task was self-paced as participants had unlimited time to respond to this question.

In the predicted reward (gold bar, gold bar) and predicted non-reward (lemon, lemon) conditions, the initial stimulus (S1) predicted (i.e., was identical to) the second stimulus (S2), which each occurred randomly on 40% of the trials. However, the task also contained 10% of unpredicted reward (lemon, gold bar) and 10% unpredicted non-reward (gold bar, lemon) trials in which S1 was a different stimulus from S2. At the end of each of the four blocks, a display appeared that showed the final account balance (i.e., “winnings”) for that block. At the conclusion of the task, participants selected one of four facedown playing cards which each corresponded a particular block “winnings" and the participant was provided the corresponding amount of cash, in addition to the cash stipend based on amount of time spent on all study procedures.

### ERP Analyses

EEG data was analyzed using Brain Vision Analyzer 2.0 using a high pass filter of 0.10 Hz and low pass filter of 40 Hz, both with a rolloff of 48 db/oct. Data was segmented from -100 to 800 ms around the onset of S2. Data was corrected for blinks and large eye movements using independent component analysis. Data was re-referenced offline to the average of all 30 scalp electrodes. Baseline correction was conducted using the interval of -100 to 0 ms prior to S2 onset. Artifact rejection removed segments within each electrode that contained more than a 120 μV deflection. Segments were then averaged for the four conditions of unexpected reward, unexpected non-reward, expected reward, and expected non-reward. Similar to previous work with this paradigm [10], we then created difference waveforms to represent better than expected outcomes (FRP and LPP components; unexpected reward minus expected non-reward) and worse than expected outcomes (FRN and LPP components; unexpected non-reward minus expected reward).

**Fig 2:**
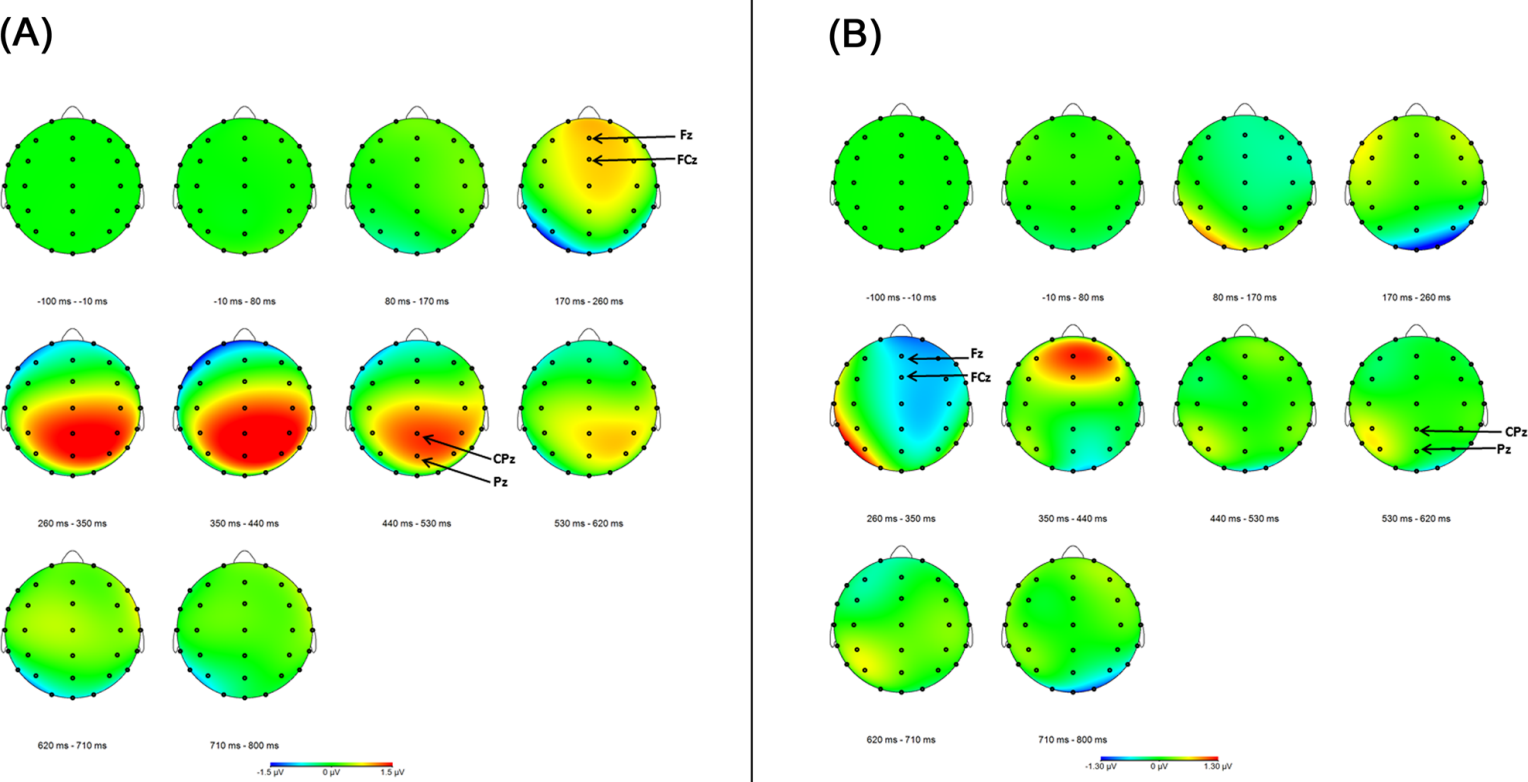
a. Grand average voltage topography maps for the better than expected outcome difference waveform (unexpected reward minus expected nonreward conditions) from the entire sample (N = 44). b. Grand average voltage topography maps for the worse than expected outcome difference waveform (unexpected nonreward minus expected reward conditions) from the entire sample (N = 44).

We examined the voltage topography plot for the better than expected difference waveform across all 44 participants. Based on the voltage topography from the 170–260 ms interval (see Fig 2A), we chose to measure the FRP component from the average of Fz and FCz electrodes. Based on this same voltage topography plot from the 350–620 ms intervals, we chose to measure the LPP component for better than expected outcomes from the average of Pz and CPz. As voltage topography from the 260–350 ms window of the worse than expected outcome condition suggested that FRN was also prominent at electrodes Fz and FCz (see Fig 2B), we chose to measure the FRN component from the average of those two electrodes which is consistent with electrodes used for FRP. In contrast to the better than expected outcome topography, there was no apparent prolonged positive voltage around Pz that would suggest a LPP response to worse than expected outcomes.

**Fig 3:**
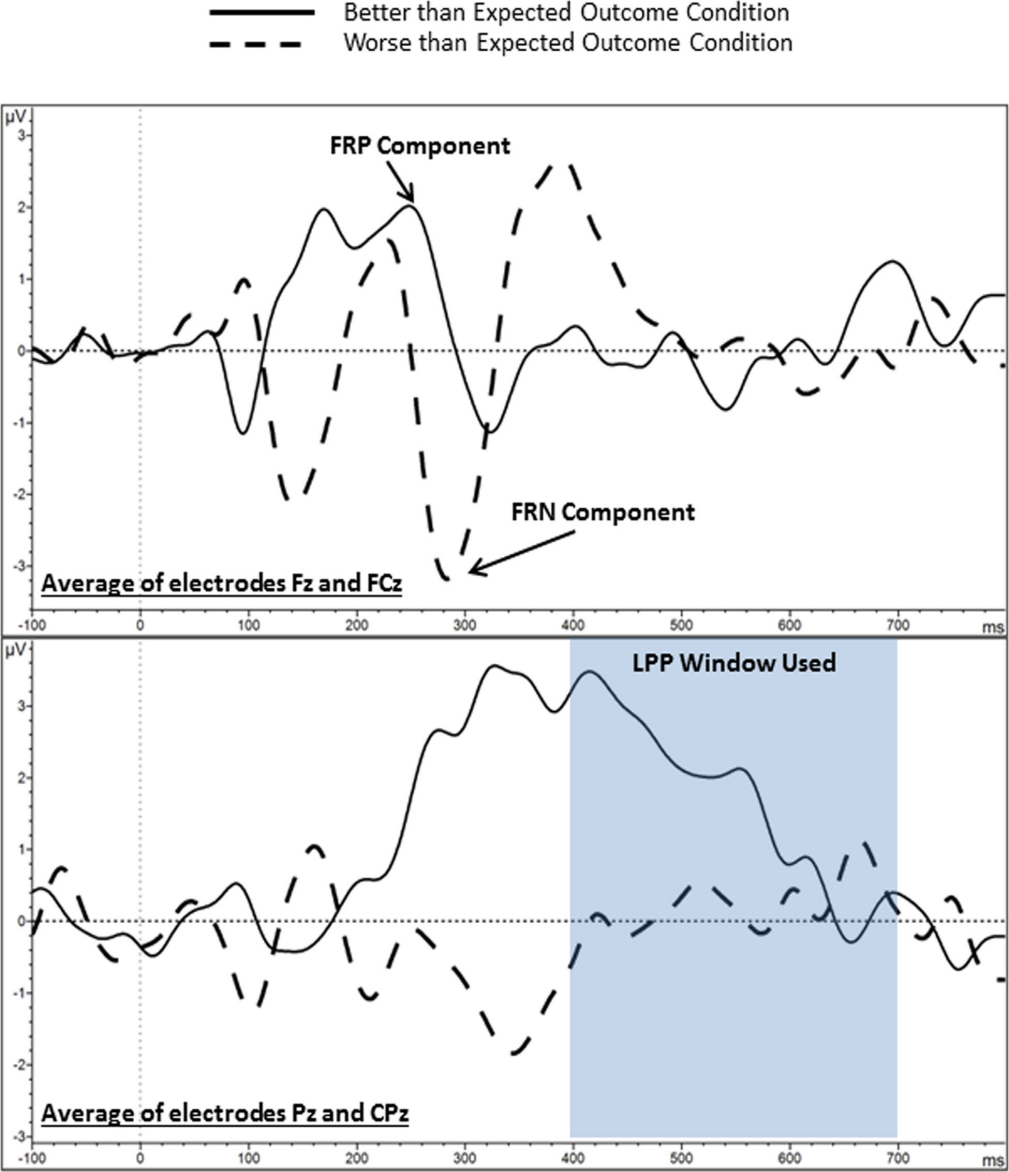
Grand average waveforms for the feedback-related positivity (FRP), feedback-related negativity (FRN), and late positive potential (LPP) components from the entire sample (N = 44). Better than expected outcome = difference waveform of unexpected reward minus expected nonreward; worse than expected outcome = difference waveform of unexpected nonreward minus expected reward. A low-pass filter of 20 Hz was used for the figure.

Consistent with the topography maps, the grand average waveforms (see Fig 3) from average of electrodes Fz and FCz to the better than expected outcomes depict FRP during better than expected outcomes (at 250 ms), and FRN during worse than expected outcomes (at 280 ms). The grand average waveforms from Pz and CPz (averaged) during better than expected outcomes show a pronounced and prolonged positive deflection from the start of the LPP range (around 400 ms) and continuing to about 700 ms, but suggest no prominent LPP to worse than expected outcomes (see Fig 3).

For each participant, we measured FRP from the better than expected outcome difference waveform, which was identified as the largest positive peak amplitude in the range of 200 to 300 ms following S2. We measured FRN from the worse than expected outcome difference waveform, which was identified as the largest negative peak amplitude in the range of 250 to 350 ms following S2. For both FRP and FRN, we extracted the mean amplitude of the peak using a 20 ms window centered on the peak latency. As LPP was only evident on the grand average waveform from the better than expected outcomes, we did not include LPP to the worse than expected outcomes in analyses. We measured LPP based on the better than expected difference waveform, using the average voltage between 400 to 700 ms following S2. This time range was based on previous literature on the LPP as well as inspection of the grand average difference waveform for better than expected outcomes (see Fig 3).

To assess the potential influence of general attention factors, we conducted a secondary analysis of the P3b component to general unexpected (i.e., rare) outcomes [41]. This was measured from a difference wave that subtracted both expected outcomes (reward and non-reward) from both unexpected outcomes. Using this difference waveform, we identified the P3b as the largest amplitude positive voltage deflection between 250 and 400 ms following S2 from the average of electrodes Pz and CPz (chosen based on existing literature [41] and inspection of voltage topography from this difference waveform). We extracted the mean amplitude of P3b using a 40 ms window centered on the peak latency.

For electrodes included in analyses of the different components (Fz, FCz, Pz, CPz), all participants had at least 95 (of 96 possible) segments that survived artifact rejection in the expected outcome conditions and all 24 segments that surved artifact rejection in the unexpected outcome conditions.

### Statistical Analysis

Pearson zero-order correlations were used to examine potential relationships between the four ERP component amplitudes and the potential confounding variables of age, sex, task accuracy, education level, and current use of nicotine (n = 12), antipsychotic medication (n = 15), sedative medication (n = 15), and/or SSRI/SNRI medication (n = 8).

We examined each of four ERP mean amplitudes of interest from the difference waveforms (FRP to better than expected outcome, FRN to worse than expected outcome, LPP to better than expected outcomes. and the P3b to both unexpected outcomes) using two approaches. The first approach used stepwise regressions (entry alpha = .05; exit alpha = .10) across the entire sample (N = 44), which included five participants with an anxiety or dysthymic disorder. The stepwise regressions were conducted seperately for each of the four ERPs of interest (FRP, FRN, LPP, and P3b), and each included nine predictors: PANSS Positive and Negative Symptom factor scores, PANSS Depression and Anxiety items from the General Factor, SPQ-BR cognitive-perceptual, interpersonal, and disorganized factor scores, TEPS total score, and ACIPS total score. There did not appear to be any problems with collinearity in any regression (Tolerance > .10, VIF < 2.5 across all predictors). According to Kolmogorov-Smirnov tests, all ERP variables had a normal distribution, but only four of the nine symptom predictors had a normal distribution. However, for all ERP and symptom variables the kurtosis and skewness was within ± 2.0. In the event of a statistically significant finding in the stepwise regressions, a separate ANCOVA was then conducted to examine whether the relationship interacted with group membership (i.e., specific to one or more of the diagnostic classes). The second, complementary, approach examined the relationship of diagnostic group membership with each ERP mean amplitude using ANCOVAs.

## Results

We excluded eight participants who had less than 75% accuracy in correctly identifying whether they won a trial within any of the four trial conditions. This resulted in 44 participants included in analyses. Examination of Pearson zero-order correlations between the eight potential confounding variables in relation to the four ERP amplitudes revealed statistically significant relationships between the P3b amplitude with age, *r*(44) = -.30, *p* = .05, and task accuracy, *r*(44) = .36, *p* = .02, as well as a relationship between the LPP voltage for better than expected outcomes and age, *r*(44) = -.33, *p* = .03. Therefore, we covaried for age and task accuracy in all remaining analyses.

**Fig 4:**
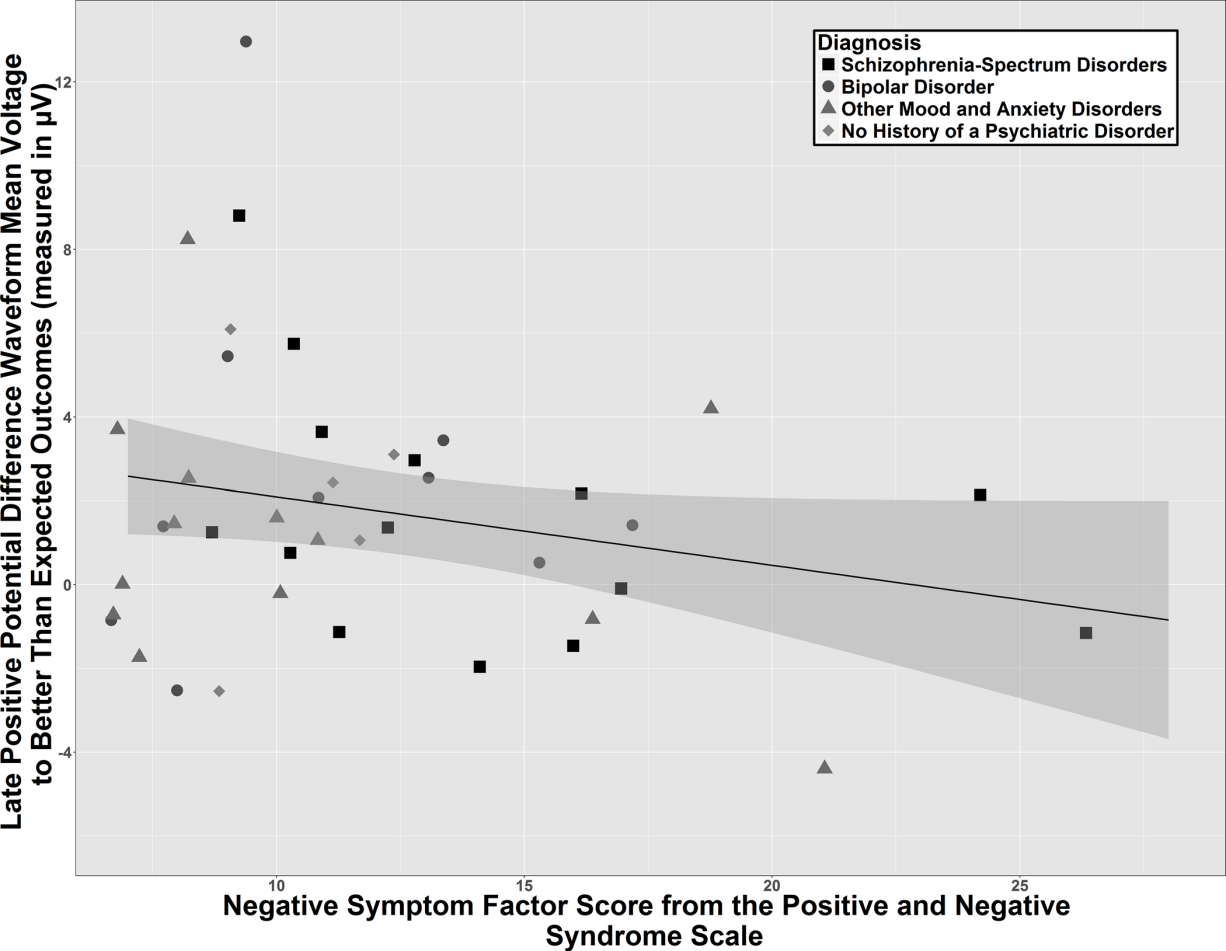
Scatterplot of the relationship of the negative symptom factor score from the positive and negative syndrome scale with the late positive potential mean voltage to better than expected outcomes across entire sample (N = 44). Better than expected outcome = difference waveform of unexpected reward minus expected nonreward.

Stepwise regressions across the entire sample revealed that the PANSS Negative Symptom factor score entered the model as a negative relationship with the LPP amplitude for better than expected outcomes, standardized β = -.30, adjusted R^2^ = .15, *t*(39) = 2.12, *p* = .04 (see Fig 4). Analysis of potential outliers revealed that one participant may be a potential outlier, as the Studentized residual was 3.29, although the Cook’s d was in acceptable range at 0.14. When this individual was removed from the overall regression, the same relationship was found and statistically significant. An ANCOVA revealed that this relationship did not interact with membership in the three diagnostic classes, suggesting that it occurred relatively independent of diagnostic status, *F*(2,31) = 0.04, *p* = .96, *η*^*2*^ = .003.

**Fig 5:**
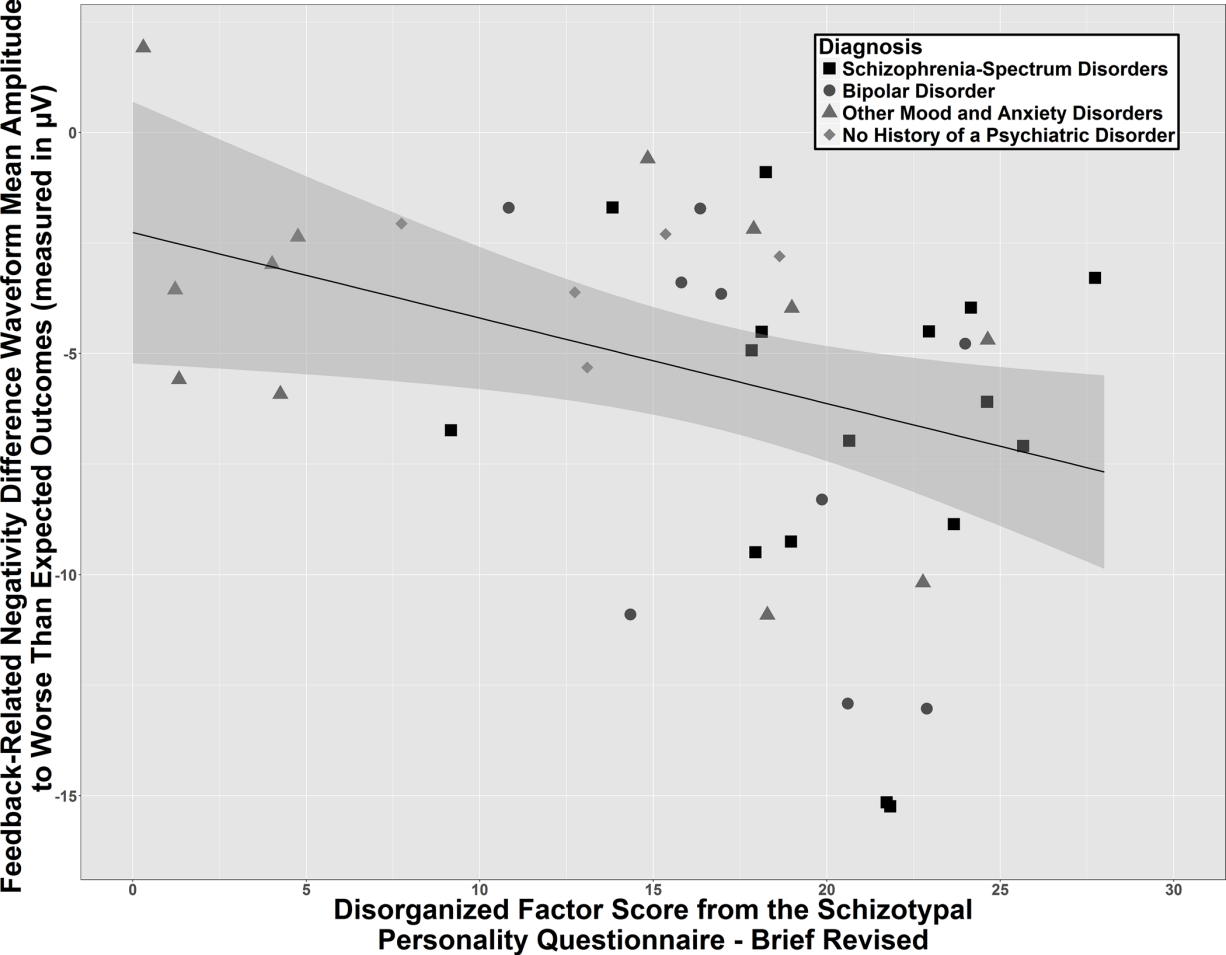
Scatterplot of the relationship of the disorganized factor score from the schizotypal personality questionnaire brief-revised with the feedback-related negativity mean amplitude to worse than expected outcomes across entire sample (N = 44). Worse than expected outcome = difference waveform of unexpected nonreward minus expected reward. One participant was missing data from this scale.

Our second exploratory stepwise regression finding was that the SPQ Disorganized factor score entered the model as a negative relationship with the FRN mean amplitude to worse than expected outcomes, standardized β = -.35, adjusted R^2^ = .10, *t*(39) = 2.42, *p* = .02. As FRN is a negative voltage waveform, this relationship translates as more severe Disorganized symptoms relating to a larger FRN mean amplitude (see Fig 5). Analysis for outliers using studentized residuals and Cook’s d revealed no potential outliers in this regression. An ANCOVA revealed that this relationship did not interact with membership in the three diagnostic classes, suggesting that it occurred relatively independent of diagnostic status, *F*(2,30) = 0.78, *p* = .47, *η*^*2*^ = .05.

No symptoms entered the stepwise regression models for the FRP or P3b mean amplitudes. When we conducted ANCOVAs to examine for differences in the ERP amplitudes across the three diagnostic classes, there was no statistically significant main effect of group for any ERP amplitude (see Table 2 for descriptive statistics by group).

**Table 2:** Peak Latencies and Mean Amplitudes of the Event-Related Potentials.

	Better Than Expected Outcome Difference Waveform*			Worse Than Expected Outcome Difference Waveform*			Unexpected Outcome Difference Waveform*	
	FRP Lat. (ms)	FRP Amp. (μV)	LPP Amp. (μV)	FRN Lat. (ms)	FRN Amp. (μV)	LPP Amp. (μV)	P3b Lat. (ms)	P3b Amp. (μV)
Entire Sample	234.34 (31.12)	34.63 (31.75)	19.60 (36.47)	290.13 (34.07)	-32.45 (29.19)	3.86 (19.01)	312.73 (34.74)	2.51 (2.73)
SSD (N = 16)	245.81 (27.15)	33.10 (35.86)	17.30 (28.84)	291.38 (36.04)	-36.24 (22.33)	6.07 (14.92)	317.13 (34.69)	1.87 (2.75)
BD (N = 10)	221.00 (34.43)	36.28 (33.66)	32.60 (51.74)	283.80 (30.82)	-45.13 (36.20)	-0.35 (27.94)	305.20 (45.22)	3.13 (2.28)
NC (N = 13)	235.23 (28.31)	32.79 (28.43)	11.02 (34.76)	280.62 (32.13)	-29.35 (29.14)	1.32 (17.97)	311.85 (34.52)	2.27 (3.20)

Descriptive statistics in format of [mean (standard deviation)]. SSD = schizophrenia-spectrum disorders; BD = bipolar disorder; NC = nonpsychiatric controls; Lat. = Peak Latency; Amp. = Mean Amplitude; FRP = feedback-related positivity; LPP = late positive potential (average voltage between 400 and 700 ms post stimulus); FRN = feedback-related negativity

* Better than expected outcome difference waveform: unexpected reward minus expected nonreward; worse than expected outcome difference waveform = unexpected nonreward minus expected reward; unexpected outcome difference = unexpected outcomes minus expected outcomes.

Note: The overall sample included an additional 5 participants with other mood and anxiety disorders.

Note: The same pattern of statistical significance in the transdiagnostic regression results were found when excluding the five participants with other mood and anxiety disorders who were not included in the group comparisons.

## Discussion

Our first hypothesis was supported, as individuals with higher Negative Symptom factor scores from the clinician-administered PANSS had a smaller voltage for the LPP from better than expected outcomes (see Fig 4). This relationship appeared to be transdiagnostic and statistically independent of the diagnostic group variable. As the LPP is thought to represent relatively more sustained affective processing from the unexpected rewards, this finding is theoretically consistent with the well-known association between negative symptoms and self-report of reduced motivation and pleasure in schizophrenia. In particular, our finding is partially consistent with a previous study with schizophrenia participants which reported that a decrease in LPP amplitude following negatively-valenced pictures related to increased negative symptoms in particular [31]. While we found this relationship with rewarding rather than aversive stimuli, the previous study did not include rewarding stimuli. Our findings suggest that a relationship between increased severity of negative symptoms and a reduction in sustained motivated attention to better than expected outcomes may extend beyond schizophrenia and represent a dimensional transdiagnostic relationship.

Our second hypothesis regarding the bipolar disorder group showing a larger FRP amplitude than both of the other groups was not supported. The three diagnostic classes did not show statistically significant differences across the ERP amplitudes and conditions. This is somewhat inconsistent with findings from a single research group in individuals with euthymic bipolar disorder [24], and two samples of individuals at psychometrically-defined risk for hypomania [25, 26], which suggested an enhanced FRP compared with nonpsychiatric controls. One possible explanation for this discrepancy is that we used a passive Pavlovian conditioning task with better and worse than expected outcomes while the Mason et al. studies used three other paradigms which did not include unexpected wins/losses based on changes to conditioned stimuli. Another possibility is Type II error due to the low statistical power resulting from our bipolar group sample size (N = 10). However, the modest effect sizes obtained when comparing the FRP amplitude from our bipolar disorder group with the schizophrenia spectrum group (*η*^*2*^ = 0.03) and nonpsychiatric control group (*η*^*2*^ = 0.08), did not suggest a strong likelihood of Type II error.

In an exploratory analysis, we found that an increased Disorganized factor score on the SPQ-BR was related to a larger FRN amplitude to worse than expected outcomes (see Fig 5). The FRN is thought to represent the relatively more immediate updating of response feedback contingencies. There does not appear to be existing literature reporting a relationship between disorganized symptoms and reactions to aversive stimuli. It is possible that individuals who have disorganized symptoms may show a greater initial orientating response to aversive stimuli to quickly avoid potentially dangerous stimuli. This may serve as a compensatory mechanism, as individuals with schizophrenia who have more severe disorganized symptoms are particularly likely to show deficits in working memory and executive functioning [42], which could interfere with more sustained threat assessment.

Finally, we examined relationships with the broader attention component of P3b to examine whether our findings with the ERPs related to emotion were driven by attention to the unexpected stimuli in general. The P3b amplitude to unexpected conditions did not show any statistically significant relationships with diagnostic class or symptoms. The P3b amplitude did show the expected negative correlation with age in our sample of adults [43] and positive correlation with task accuracy (i.e., index of attention to task), suggesting that it was reliably elicited and measured. This suggests that our symptom findings with LPP and FRN were due to the emotional salience of the stimuli rather than general attention-related factors.

Our study was limited by a relatively small sample size and the inability to examine reward and loss anticipation (e.g., the stimulus preceding negativity ERP component) based on the task design. In addition, the use of linked mastoid electrodes as the active reference during recording is not ideal as this can bias estimates of laterality in the signal [44]. To partially address this limitation, we re-referenced to the common average offline and restricted our analyses to pairs of midline electrodes where these components have been well-established in the existing literature. However, a strength of the study is the use of a broad transdiagnostic sample and analytic approach, along with examination of three different types of reward ERPs to both rewarding and aversive outcomes in the same paradigm.

While our transdiagnostic findings were partially exploratory, they create a testable model for future studies using similar transdiagnostic approaches to examine the neurophysiology of reward processing abnormalities. Specifically, other studies can test the theory that greater severity of disorganized symptoms, regardless of diagnosis, relate to a greater FRN amplitude following worse than expected outcomes, or more broadly to exaggerated indices of immediate expectation processing of aversive outcomes. Similarly, other studies can examine whether greater severity of negative symptoms, regardless of diagnosis, relate to a reduced LPP following better than expected outcomes, or more generally a reduction in sustained motivated attention to and processing of rewards. If replicated, these findings can directly inform treatment efforts by helping clinicians target particular abnormalities in reward/aversion processing based on individual patient characteristics such as relative severity of disorganized and negative symptoms, regardless of the disorder the patient is presenting with.
